# Unraveling the Mystery: A Report of a Rare Case of Whipple's Disease Diagnosed After Months of Unexplained Symptoms

**DOI:** 10.7759/cureus.81700

**Published:** 2025-04-04

**Authors:** Ana Filipa Martins, Cleide Moreira, Inês de Brito Gonçalves, Alexandra Mendes, Rita Matos Sousa

**Affiliations:** 1 Internal Medicine, Hospital de Braga, Braga, PRT; 2 Pulmonology, Hospital de Braga, Braga, PRT; 3 Clinical Sciences, Escola de Medicina Universidade do Minho, Braga, PRT

**Keywords:** anemia, case report, diarrhea, infectious disease, weight loss, whipple's disease

## Abstract

Whipple's disease is a rare, chronic infectious condition with a highly variable presentation, often manifesting as arthralgia, diarrhea, and abdominal pain, resembling other gastrointestinal and rheumatologic disorders. As a result, diagnosis is often challenging and frequently delayed for three to seven years after symptom onset. Here, we present a case of a 61-year-old man who experienced abdominal distension, nausea, 18 kg weight loss (25% body weight), and diarrhea for six months. After an extensive workup, including endoscopies, imaging, and laboratory tests, a diagnosis of Whipple's disease was confirmed based on duodenal biopsy showing periodic acid-Schiff (PAS)-positive macrophages. The diagnostic process took three months, underscoring the difficulty in identifying this rare, unusual condition. Treatment was guided by more recent discoveries regarding the susceptibility of *Tropheryma whipplei. *This case highlights the importance of maintaining a high clinical suspicion of Whipple's disease in patients with unexplained symptoms.

## Introduction

Whipple's disease (WD) is a rare systemic infection caused by *Tropheryma whipplei,* and its current prevalence is yet to be determined. WD predominantly affects middle-aged Caucasian men, but, although less common, can occur in individuals of any age [1,2].

Its clinical presentation is highly variable, though a characteristic tetrad of arthralgia, diarrhea, abdominal pain, and weight loss is commonly described [3]. However, nearly one-third of the patients lack gastrointestinal symptoms and instead present with multisystemic manifestations [4]. Arthralgia and fever often appear as initial symptoms, followed by gastrointestinal involvement and, in advanced stages, potential neurological complications [5,6].

Diagnosis of WD remains a challenge, with an average delay of three to seven years between symptom onset and diagnosis [5]. This delay is largely attributed to the disease's rarity and its non-specific presentations, leading to frequent misdiagnosis as rheumatologic or gastrointestinal disorders [7,8]. Due to the potential severity of WD, physicians must maintain a high index of suspicion, particularly in patients presenting with unexplained weight loss, chronic diarrhea, and refractory arthralgias despite standard therapy.

A definitive diagnosis is typically established through a duodenal biopsy showing periodic acid-Schiff (PAS)-positive macrophages in the lamina propria, with polymerase chain reaction (PCR) testing of intestinal biopsy samples serving as a valuable adjunct, demonstrating >90% sensitivity and specificity [9]. However, positive PCR results from saliva, stool, or joint fluid alone have limited positive predictive value, as *Tropheryma whipplei* can be present in asymptomatic carriers.

Prognosis largely depends on early diagnosis and treatment. While most cases respond well to early antibiotic therapy, relapse - defined as the return of symptoms after initial remission or improvement, often due to inadequate treatment, antibiotic resistance, or persistent infection - occurs in up to 35% of cases [10]. Additionally, immune reconstitution inflammatory syndrome can develop in some patients undergoing treatment, necessitating careful monitoring [11].

## Case presentation

A 61-year-old male, unemployed for 15 years due to chronic low back pain, presented to the emergency department with persistent complaints of abdominal distension, nausea, anorexia, postprandial fullness, and an 18 kg weight loss (25% body weight) over the past six months. He experienced two daily episodes of watery, non-bloody, non-mucoid diarrhea but denied fever, vomiting, skin rash, or additional joint pain. On admission, the patient exhibited pale mucous membranes, skin hyperpigmentation, and a severely emaciated appearance. Laboratory findings revealed acute microcytic hypochromic anemia due to iron deficiency (hemoglobin 6.8 g/dL, hematocrit 21.2%), hypoalbuminemia, and normal renal, liver, and thyroid functions. Infectious serology, including HIV testing, was negative. Thoracoabdominal imaging revealed several dense mesenteric lymphadenopathies of unknown significance.

The patient was admitted to the internal medicine department for urgent etiological evaluation. The differential diagnoses considered included functional gastrointestinal disorders, inflammatory bowel disease (given the second peak of incidence), microscopic colitis (despite the infrequent bowel movements), chronic pancreatitis, food intolerance (such as celiac disease), chronic intestinal infection, gastric lymphoma, and systemic rheumatic disease due to the presence of arthralgias.

**Figure 1:**
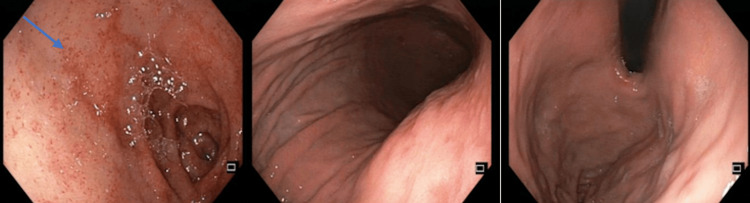
First upper digestive endoscopy performed at the beginning of hospitalization This examination reveals an unchanged esophageal lumen and an unremarkable mucosa in the body, fundus, and antrum. However, the duodenal bulb exhibits punctate hemorrhagic lesions (arrow).

For diagnostic assessment, stool samples were collected for parasitological and microbiological analysis. Additional testing included immunologic markers (erythrocyte sedimentation rate, anti-endomysial antibody, anti-tissue transglutaminase antibodies, antinuclear antibodies, anti-double-stranded DNA, antineutrophil cytoplasmic antibodies, rheumatoid factor, serum protein electrophoresis, cryoglobulins) and infectious serologies (viral hepatitis panel, cytomegalovirus [CMV], herpes I and II, Epstein-Barr virus (EBV), parvovirus B19). Upper gastrointestinal endoscopy revealed scattered punctate hemorrhagic lesions in the duodenal bulb (Figure 1); however, no biopsies were taken, reducing the diagnostic value of this test. Lower gastrointestinal endoscopy had a normal mucosal appearance. Abdominal MRI showed lymphadenopathy consistent with celiac sprue. Chronic pancreatitis was ruled out based on normal imaging findings and a normal fecal elastase level.

The patient was followed up in an outpatient medical clinic with a gluten-free diet and oral iron supplementation. Despite celiac disease being one of the differential diagnoses for the symptoms, the probability was low since there were no positive anti-endomysial antibodies, anti-tissue transglutaminase antibodies, or HLA-DQ2/DQ8 positivity. A video capsule endoscopy revealed diffuse edema and multiple lymphangiectasias, and erythematous areas affecting the villi in the duodenum and jejunum.

**Figure 2:**
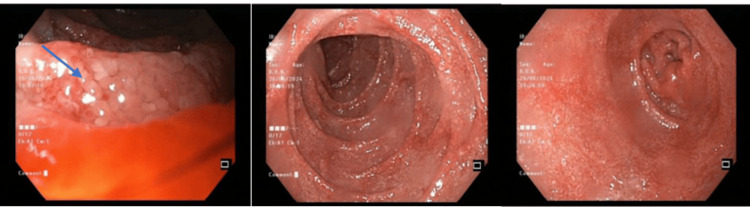
Second upper digestive endoscopy performed three months after the patient's first evaluation This examination reveals a normal esophageal lumen and mucosa, with the gastric mucosa in the fundus, body, and antrum showing areas of erythema and erosions. The mucosa of the duodenal bulb and the second portion of the duodenum (D2) exhibits edema of the villi (arrow), lymphangiectasias, and petechiae, in a diffuse pattern.

Because of the persistent symptoms and the absence of a definitive diagnosis, endoscopic studies were repeated with biopsies. Upper gastrointestinal endoscopy revealed numerous petechial hemorrhages and diffuse villous edema with lymphangiectasias in the duodenum (Figure 2). WD was finally diagnosed through histopathological findings of PAS-positive macrophages in duodenal biopsies, confirming the diagnosis three months post-admission. *Tropheryma whipplei* DNA was also detected in a stool sample.

Despite the lack of neurological symptoms, lumbar puncture was also performed to exclude central nervous system involvement (with PCR testing for *Tropheryma whipplei* DNA returning negative). The patient was started on intravenous ceftriaxone at a dose of 2 g daily for two weeks, followed by doxycycline 100 mg twice daily and hydroxychloroquine 200 mg three times daily. On follow-up, there was an early indication of good response to treatment. At six months, he was asymptomatic, leading a more active life without requiring assistance for walking. He had gained 12 kg (22% of his body weight), and his hemogram was normal, with a hemoglobin level of 15.9 g/dL and a hematocrit of 46.3%.

## Discussion

WD is a challenging diagnosis because of its rarity and broad variability in clinical presentation. The time from symptom onset to diagnosis is, on average, three to seven years [5]. This delay can be attributed to several factors, such as low disease prevalence, nonspecific presentation, and frequent misdiagnosis as another condition. Most significantly, roughly 50% of patients are initially misdiagnosed with a rheumatologic inflammatory disease [5]. This disease often mimics more common conditions such as inflammatory bowel disease, connective tissue disease, and other infections [7].

This case report underscores the diagnostic challenges of WD, which took three months despite an extensive workup. Multiple differential diagnoses were considered, and the diagnostic workup was extremely thorough with numerous tests, including stool samples, immunological markers, infectious serologies, upper and lower gastrointestinal endoscopies, abdominal MRI, and video capsule endoscopy.

One of the significant difficulties was the nonspecific nature of the symptoms. In this case, the patient had a new onset of constitutional symptoms over a period of six months, with chronic back pain that had possibly been overlooked or undertreated. Any worsening of chronic pain may be one of the early indications of the infection, as evidenced by the patient’s improvement after treatment.

Another challenge was the delay in taking biopsies during the first endoscopic examination, mainly because macroscopic abnormalities in the duodenal mucosa were not initially evident. In this context, video capsule endoscopy played a crucial role in prompting early repeat endoscopic evaluations, particularly given the chronic nature of the symptoms, ultimately aiding in establishing a definitive diagnosis. Capsule endoscopy is a valuable tool in diagnosing WD, offering several key advantages. It allows complete visualization of the entire small intestine through a less invasive approach compared to conventional endoscopy. It is particularly useful when pathology is present in the distal small bowel, beyond the reach of standard gastroscopy. The technique also facilitates complete disease mapping, allowing a comprehensive assessment of the extent and severity of intestinal involvement [10].

Once a confirmatory diagnosis is established by histopathological examination of PAS-positive macrophages in the lamina propria, appropriate treatment must be initiated promptly. The importance of ruling out central nervous system (CNS) involvement in WD cannot be overstated. This case report supports the approach of performing a lumbar puncture even in the absence of neurological symptoms, highlighting the proactive stance required in managing the disease [5]. CNS involvement is often asymptomatic and can only be diagnosed through PCR detection of *T. whipplei* in the cerebrospinal fluid. Among patients with symptomatic CNS involvement, cognitive dysfunction, including dementia, memory impairment, and confusion, is the most common abnormality.

Treatment of WD is typically a two-stage process. The initial stage involves intravenous antibiotics, most commonly ceftriaxone, for two to four weeks. This is followed by long-term oral antibiotics, most commonly trimethoprim-sulfamethoxazole, which must be taken for at least 12 months. As evident from the case report, other combinations such as doxycycline and hydroxychloroquine may also be utilized. WD treatment has dramatically changed since the initial culture of *T. whipplei*. Even though classical treatment with trimethoprim-sulfamethoxazole is confronted with increasing failure rates, the combination of doxycycline and hydroxychloroquine has emerged as an attractive alternative [12]. Remarkably, *T. whipplei *lacks the gene encoding for dihydrofolate reductase, the target enzyme for trimethoprim, and this component might, therefore, be potentially ineffective [12]. The combination of doxycycline and hydroxychloroquine has a two-pronged mechanism of action: hydroxychloroquine increases the pH of phagocytic vacuoles, where *T. whipplei* thrives, thereby enhancing doxycycline’s antimicrobial activity in the now more alkaline environment [13]. Recent clinical studies have shown that an entirely oral regimen of doxycycline and hydroxychloroquine is as effective as intravenous ceftriaxone plus trimethoprim-sulfamethoxazole, with the added benefits of shorter hospital stays and improved patient adherence [13]. An increasing trend has emerged in favor of a one-year combination treatment of doxycycline and hydroxychloroquine, followed by a personalized evaluation to determine the need for ongoing prophylactic therapy [12,13]. Early diagnosis and appropriate antibiotic treatment usually result in good outcomes; however, relapse can occur in a significant percentage of patients, necessitating close follow-up [10].

## Conclusions

The rarity of this condition makes differential diagnosis complex and broad, requiring a high index of suspicion and extensive investigation once more common conditions have been excluded. This case highlights the importance of persisting in diagnostic efforts in the setting of unexplained symptoms, particularly in the presence of the classical tetrad of abdominal pain, arthralgia, diarrhea, and weight loss. The three-month diagnostic timeline, which involved multiple investigative modalities and serial endoscopies, highlights the challenge for clinicians in diagnosing WD. Early detection and timely treatment are crucial for achieving optimal patient outcomes.
